# Pulmonary Haemodynamics in Sickle Cell Disease Are Driven Predominantly by a High-Output State Rather Than Elevated Pulmonary Vascular Resistance: A Prospective 3-Dimensional Echocardiography/Doppler Study

**DOI:** 10.1371/journal.pone.0135472

**Published:** 2015-08-13

**Authors:** Sitali Mushemi-Blake, Narbeh Melikian, Emma Drasar, Amit Bhan, Alan Lunt, Sujal R. Desai, Anne Greenough, Mark J. Monaghan, Swee Lay Thein, Ajay M. Shah

**Affiliations:** 1 King’s College London British Heart Foundation Centre, Cardiovascular Division, London, United Kingdom; 2 Department of Cardiology, King’s College Hospital, Denmark Hill, London, United Kingdom; 3 Department of Haematological Medicine, King’s College Hospital, Denmark Hill, London, United Kingdom; 4 Department of Paediatrics, King’s College Hospital, Denmark Hill, London, United Kingdom; 5 Department of Radiology, King’s College Hospital, Denmark Hill, London, United Kingdom; 6 NIH / National Institute of Heart, Lung and Blood Diseases, Sickle Cell Branch, Bethesda, Maryland, United States of America; Université Claude Bernard Lyon 1, FRANCE

## Abstract

**Aims:**

Patients with sickle cell disease have significant morbidity and mortality. Pulmonary hypertension is suggested to be an important contributor but its nature and severity in these patients and how best to non-invasively assess it are controversial. We hypothesised that a high-output state rather than primary pulmonary vascular pathology may be the major abnormality in sickle cell disease. This study aimed to evaluate the characteristics and severity of pulmonary hypertension in patients with sickle cell disease using detailed echocardiography.

**Methods and Results:**

We undertook a prospective study in 122 consecutive stable outpatients with sickle cell disease and 30 age, gender and ethnicity-matched healthy controls. Echocardiographic evaluation included 3D ventricular volumes, sphericity, tissue Doppler, and non-invasive estimation of pulmonary vascular resistance. 36% of patients had a tricuspid regurgitant velocity ≥2.5 m.s^-1^ but only 2% had elevated pulmonary vascular resistance and the prevalence of right ventricular dysfunction was very low. Patients with raised tricuspid regurgitant velocity had significantly elevated biventricular volumes and globular left ventricular remodelling, related primarily to anaemia. In a subgroup of patients who underwent cardiac catheterization, invasive pulmonary haemodynamics confirmed the echocardiographic findings.

**Conclusions:**

Elevated cardiac output and left ventricular volume overload secondary to chronic anaemia may be the dominant factor responsible for abnormal cardiopulmonary haemodynamics in patients with sickle cell disease. 3D echocardiography with non-invasive estimation of pulmonary vascular resistance represents a valuable approach for initial evaluation of cardiopulmonary haemodynamics in sickle cell disease.

## Introduction

Sickle cell disease (SCD), prevalent in peoples of African descent, results from the presence of haemoglobin S (HbS) due to a genetic mutation in the β-globin chain of haemoglobin. The abnormal HbS polymerizes under low oxygen conditions leading to the formation of irreversibly sickled red blood cells that cause repeated episodes of vaso-occlusion and chronic anaemia, with multi-organ complications that impose substantial morbidity and reduce life expectancy [1]. With improved general medical care and reduction in infective complications, SCD has evolved into a chronic condition in which current treatment options are largely limited to blood transfusion and hydroxyurea [2].

Considerable attention has focused on pulmonary arterial hypertension as a potential cause of long-term morbidity and mortality in SCD [3–6]. It was proposed that chronic haemolysis leads to depletion of vasodilator nitric oxide in the microcirculation and induces an increase in pulmonary resistance that has long-term detrimental effects [4]. These authors used echocardiographically-measured tricuspid valve regurgitation velocity (TRV) of ≥2.5 m.s^-1^ as a surrogate marker of abnormally elevated pulmonary arterial pressure and reported that >30% of patients had abnormal values and that this correlated with an increased likelihood of premature death [4]. Other studies also found a high prevalence of TRV ≥2.5 m.s^-1^ in SCD [7,8]. However, this hypothesis has been challenged (6), and clinical trials of agents that target pulmonary arterial hypertension have been disappointing in SCD [9,10]. More recent studies involving systematic right heart catheterisation in patients with SCD and elevated TRV suggest a much lower prevalence of pulmonary hypertension [5,11]. In the largest study, Parent et al [5] found only 6% of patients to have pulmonary hypertension and this was most commonly post-capillary (venous) rather than pre-capillary (arterial). These authors concluded that simple echocardiographic evaluation alone is of limited value for the detection of pulmonary hypertension in SCD. However, invasive and potentially repeated assessment by right heart catheterisation in all patients is impractical. Furthermore, the pathophysiology responsible for the high prevalence of elevated TRV in stable patients with SCD remains unclear.

Most screening studies in SCD to date have employed basic 2D Doppler echocardiography and have not taken advantage of techniques such as 3D imaging (which allows accurate volume estimation), tissue Doppler, strain analysis and non-invasive estimation of pulmonary vascular resistance (PVR). Cardiac output in SCD is often significantly elevated secondary to chronic anaemia and may confound interpretation of TRV. We hypothesized that elevated cardiac output rather than an elevated PVR may be the major driver of abnormal cardiopulmonary haemodynamics in SCD and that the relative contributions of these factors can be assessed by comprehensive echocardiography. The primary aim of this study was to prospectively undertake comprehensive non-invasive evaluation of cardiopulmonary haemodynamics in a large population of outpatients with SCD and stable symptoms.

## Methods

### Study population

We studied 152 subjects, comprising 122 consecutive adult outpatients with stable SCD and 30 healthy controls matched for age, gender and ethnicity. Patients with a painful sickle crisis within the previous 6 weeks were excluded. The SCD genotype was haemoglobin SS in 82 (67%), haemoglobin SC in 22 (18%) and haemoglobin S-beta thalassaemia in 18 (15%). Controls were recruited from the general public and hospital staff. The study complied with the Declaration of Helsinki and was approved by the National Research Ethics Service London Dulwich Committee and the King’s College Hospital Directorate of Research and Development. All participants provided written informed consent.

### Echocardiography

Transthoracic echocardiography was performed according to American Society of Echocardiography guidelines [12], using a Philips IE33 system with 2.5 MHz matrix array and stand-alone transducers. TRV was measured with continuous wave Doppler, using the highest value obtained in any of four standard views. Non-invasive estimation of PVR (i.e. PVR_echo_) was derived from TRV and the RV outflow tract time-velocity integral (PVR_echo_ = 10 x TRV / RV outflow tract time-velocity integral), as previously described [13]. 2D colour-coded pulse wave tissue Doppler recordings were made at the level of the LV lateral mitral valve annulus and the RV free wall tricuspid valve annulus. The ratios of early diastolic LV inflow (E) to lateral mitral annulus velocity (E’), and RV inflow to tricuspid free wall annulus velocity, were derived as measures of LV and RV filling pressure respectively [14]. Tricuspid annular plane systolic excursion (TAPSE) was recorded as a measure of right ventricular systolic function [15]. Images were analysed off-line using Philips Xcelera software.

LV myocardial strain analysis was performed using standard grayscale 2D images in the short axis parasternal view at the level of the papillary muscle and in the 4-chamber apical view. Myocardial deformation was quantified at basal, mid and apical levels by speckle tracking analysis [16]. Strain quantification was performed using Philips QLab software v.9.

Full-volume 3D datasets were obtained from the apical 4-chamber window over 4 consecutive cardiac cycles and analyzed off-line [17]. 3D sphericity index was calculated as LV end-diastolic volume divided by volume of a sphere with the same diameter as the LV long-axis length; a higher index indicates a more globular LV. LV remodelling index was calculated as the ratio of LV mass to LV end-diastolic volume; it is usually unchanged in physiological hypertrophy, increased in pathological hypertrophy and decreased in heart failure [18].

### Cardiac catheterisation and pulmonary assessment

A random subset of 18 patients with a TRV ≥2.5 m.s^-1^ underwent right heart catheterization, pulmonary high resolution computed tomography, and lung function testing, performed within a 2-week window for each patient. Measurements were made of pulmonary artery pressure (PAP), pulmonary capillary wedge pressure (PCWP) and thermodilution cardiac output. Pulmonary vascular resistance (PVR_RHC_) was calculated in Wood Units. Computed tomography was performed on a dual detector helical scanner (HiSpeed NX/I, GE Medical Systems, Milwaukee, WI). Lung function tests were assessed according to American Thoracic Society/European Thoracic Society guidelines [19].

#### Laboratory tests

Routine haematology and biochemistry profiles were performed in the local hospital laboratories.

#### Statistics

Data are presented as mean±SD or percentage as appropriate. Student's t-test was used to compare continuous variables and Chi square or Mann-Whitney U test to compare dichotomous variables. Associations between continuous variables were quantified by Pearson’s correlation coefficients (two-tailed). Multivariable regression models with stepwise elimination, based on a selection of clinical and laboratory variables, were created to determine the independent determinants of echocardiographic parameters. Standardized (beta) coefficients were calculated for covariates in models. Data analyses were performed using SPSS statistical software. P<0.05 was considered significant.

## Results

### Patient characteristics

All patients and controls were of African-Caribbean or African ethnic origin, with a majority of females. The SCD cohort had typical clinical characteristics of the disease, including multi-organ complications, chronic anaemia, reticulocytosis and mild hypoxaemia (Table 1). 62% of all SCD outpatients had at least 1 sickle-related complication. Blood pressure was significantly lower in patients with SCD.

**Table 1:** Clinical characteristics of SCD patients compared with matched control subjects.

Patient characteristic	Controls	SCD	P value
Number (%)	30	122	
Age, yrs	38±9	37±14	ns
Female (%)	18 (60)	79 (65)	ns
Body surface area, m^2^	2.0±0.2	1.7±0.2	<0.01
Systolic blood pressure, mmHg	123±15	115±14	0.01
Diastolic blood pressure, mmHg	76±10	68±10	<0.01
Oxygen saturation, %	98±3	95±4	<0.01
>2 emergency department visits in past 5 yrs (%)	-	40 (35)	
History of respiratory disease (%)	-	20 (16)	
Controlled systemic hypertension (%)	-	16 (13)	
History of liver disease (%)	-	10 (8)	
History of pulmonary embolism (%)	-	10 (8)	
History of stroke (%)	-	9 (7)	
History of renal dysfunction (%)	-	9 (7)	
Hydroxyurea therapy (%)	-	18 (15)	
Regular transfusion programme (%)	-	14 (11)	
Haemoglobin, g/dl	13±1.1	9.2±2.0	<0.01
White cell count, 1000/μl	7.4±3.0	9.5±3.9	<0.01
Platelet count, 1000/μl	252±66	355±162	<0.01
Reticulocyte count, x10^-3^/ μl (normal range 50–150)	-	306±141	
Lactate dehydrogenase, IU/L (normal <240)	-	371±143	
Bilirubin, μmol/L (normal range 3–20)	-	56±71	
Asparate aminotransferase, IU/L (normal range 10–50)	-	44±22	
Alkaline phosphatase, IU/L (normal range 30–130)	-	92±46	
Blood urea nitrogen, mmol/L (normal range 3.3–6.7)	-	12±7.6	
Creatinine, μmol/L (normal range 45–120)	-	82±68	

### Non-invasive assessment of pulmonary haemodynamics

**Fig 1:**
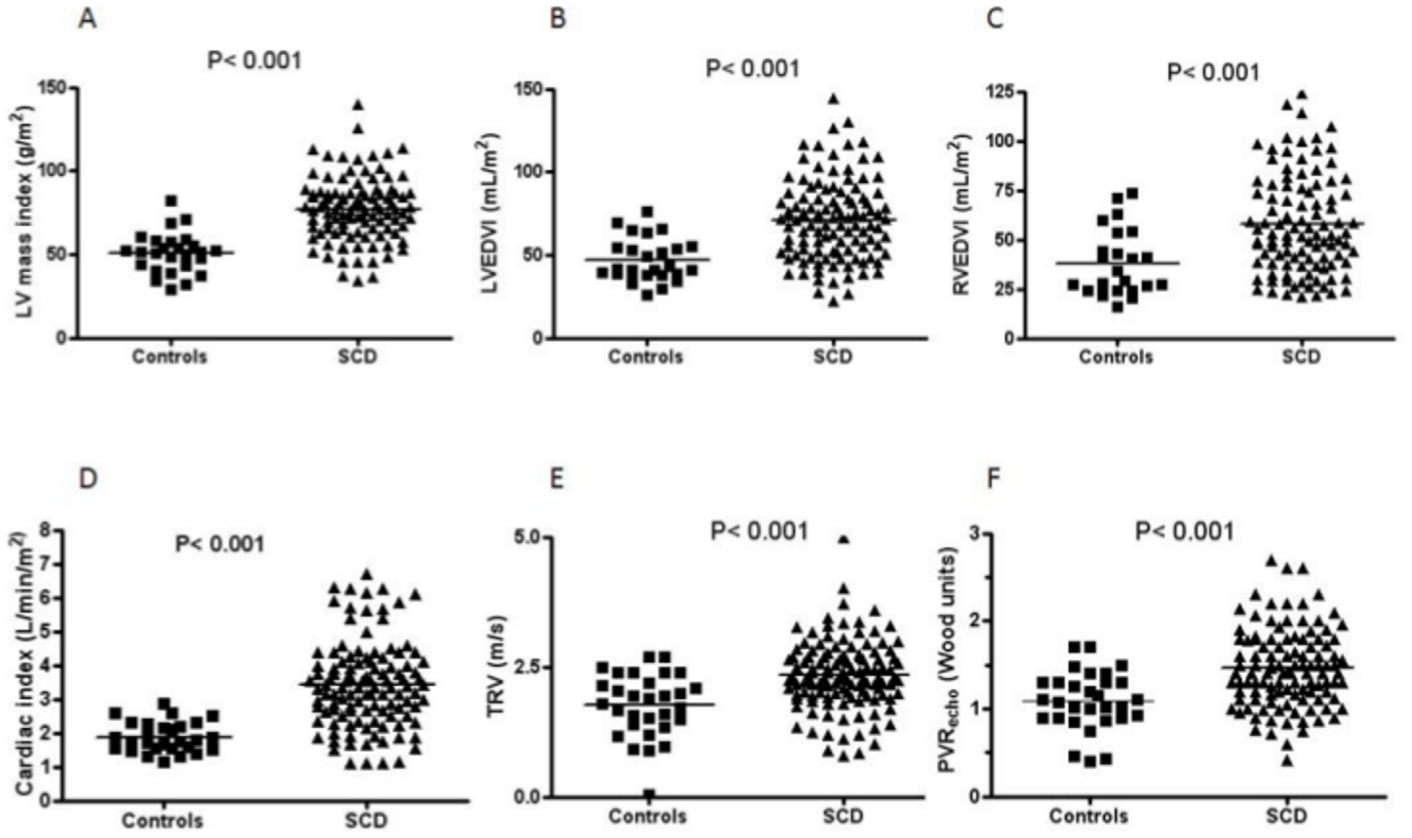
Scatter plots showing the distribution of values for echocardiographic parameters in SCD and control subjects. LVEDVI, LV end-diastolic volume index; RVEDVI, RV end-diastolic volume index.

Patients with SCD had significantly higher TRV and PVR_echo_ values than control subjects (Fig 1, Table 2). However, although 36% of patients had a TRV ≥2.5 m.s^-1^ and 12% had a TRV ≥3 m.s^-1^, only 2% had a PVR_echo_ >3 Wood units, which is the accepted cutoff for an abnormally elevated PVR [20]. 14% of patients had a PVR_echo_ between 2–3 Wood units.

**Table 2:** Echocardiographic parameters in SCD outpatients.

	Controls (n = 30)	SCD (n = 122)	P value
Tricuspid regurgitation velocity, m/s	1.8±0.6	2.3±0.6	<0.01
PVR_echo_, Wood units	1.0±0.3	1.5±0.6	<0.01
RV ejection fraction, %	53±4	62±8	0.02
TAPSE, cm	2.1±0.4	2.4±0.4	<0.01
RV end-diastolic volume index, mL/m^2^	38±16	58±25	<0.01
RV end-systolic volume index, mL/m^2^	14±9	22±11	<0.01
RV free wall E/E’ ratio	5.3±1.7	5.6±2.1	0.55
LV end-diastolic volume index, mL/m^2^	47±12	71±24	<0.01
LV end-systolic volume index, mL/m^2^	19±8	28±10	<0.01
LV ejection fraction, %	59±6	61±7	0.12
Cardiac index, L/min/m^2^	1.9±0.4	3.4±1.3	<0.01
LV stroke work index, g/m/m^2^	3.5±0.9	5.2±1.8	<0.01
LV lateral E/E’ ratio	6.9±3	7.3±2	0.55
LV mass index, g/m^2^	50±13	75±23	<0.01
Sphericity index diastole, %	26±5	33±6	<0.01
Sphericity index systole, %	21±5	26±6	<0.01
LV remodelling index, g/ml	1.1±0.3	1.1±0.4	0.49

To assess the functional impact of potential pulmonary hypertension, we assessed RV structure and function. Both RV ejection fraction and TAPSE were significantly higher in patients than controls, suggesting preserved or increased systolic function (Table 2). RV diastolic function assessed by tricuspid E/E’ was similar in patients and controls. The 3D RV end-diastolic and end-systolic volumes were significantly higher in SCD than controls (Fig 1, Table 2). These results suggest that the elevated TVR values are not related to a raised PVR_echo_ but patients with SCD have an enlarged RV.

### Left ventricular structure and function

Pulmonary haemodynamics may also be influenced by left-sided heart pressures and volumes, which can contribute to pulmonary venous hypertension. Patients with SCD had a significantly higher 3D cardiac index, LV end-diastolic and end-systolic volume index than controls (Fig 1, Table 2). The main independent determinants of LV end-diastolic volume on multivariable regression analysis were haemoglobin concentration (P = 0.04) and female gender (P = 0.01). LV mass index was also significantly higher in SCD. The 3D LV sphericity index was significantly higher in SCD (Table 2), indicating globular LV remodelling. Ejection fraction and LV remodelling index were unaltered in SCD. LV stroke work index, which controls for differences in afterload, was significantly higher in patients than controls. The LV E/E' ratio was similar in patients and controls.

We undertook analyses of LV strain, which provide a less load-dependent measure of systolic function [16], in a subgroup of 22 patients with the most enlarged hearts (all with the haemoglobin SS genotype). This showed no significant differences between SCD and control groups (S1 Table). Taken together, these results indicate that patients with SCD have a volume-overloaded LV with compensated LV hypertrophy and preserved systolic function.

### Determinants of elevated TRV

Table 3 compares the characteristics of SCD patients with TRV ≥2.5 m.s^-1^ and <2.5 m.s^-1^. Patients with elevated TRV had significantly lower haemoglobin levels and oxygen saturation, higher LDH levels and cardiac volumes, and were more likely to have suffered previous pulmonary embolism. RV systolic function assessed by TAPSE was more hyperdynamic in patients with TRV ≥2.5 m.s^-1^.

**Table 3:** Clinical, laboratory and echocardiographic parameters for patients divided according to TRV <2.5 and ≥2.5 m/s. eGFR, estimated glomerular filtration rate.

	TRV <2.5 m/s	TRV ≥2.5 m/s	P value
**Clinical parameter**			
Number of patients	78	44	
Age, years	35±13	39±14	0.1
Female gender (%)	49(64)	30(65)	0.93
Pulmonary embolism (%)	3 (4)	7 (15)	0.03
Regular blood transfusion (%)	6 (7)	8 (17)	0.11
Hydroxyurea therapy (%)	10 (13)	8 (17)	0.53
Mean blood pressure, mmHg	85±11	82±11	0.24
Oxygen saturation, %	96±3	94±5	0.02
**Laboratory markers**			
Haemoglobin, g/dl	9.9±2.0	8.1±1.6	<0.01
Reticulocyte count, x10^-3^/ μl	303±151	309±127	0.81
Lactate dehydrogenase, IU/l	326±117	441±153	<0.01
Bilirubin, μmol/l	50±38	50±33	0.97
eGFR, ml/min	87±29	90±28	0.74
Proteinuria (%)	13 (17)	21 (45)	<0.01
**Echocardiographic parameters**			
TAPSE, cm	2.3±0.4	2.5±0.4	0.01
RV free wall E/E’ ratio	5.4±1.8	5.8±2.4	0.43
Cardiac index, L/min/m^2^	3.2±1.2	3.7±1.2	0.06
LV end-diastolic volume index, ml/m^2^	67 ± 22	79 ± 26	0.01
LV ejection fraction, %	61±6	61±8	0.92
LV lateral wall E/E’ ratio	6.9±2.2	7.9±2.5	0.06

We looked for association between TRV and various clinical, laboratory and echocardiographic parameters and then undertook multivariable regression analysis for independent determinants of TRV ≥2.5 m.s^-1^. This showed that haemoglobin concentration and a history of pulmonary embolism were independent determinants of TRV ≥2.5 m.s^-1^ (S2 Table). Since very few patients had PVR_echo_ ≥3 Wood units, we undertook a similar analysis in those with PVR_echo_ ≥2 Wood units (22 patients; 18% of the total cohort). On multivariable regression analysis, the independent determinants of PVR_echo_ ≥2 Wood units were a history of pulmonary embolism and advancing age.

### Invasive assessment of pulmonary haemodynamics

The findings so far suggest that elevated TRV ≥2.5 m.s^-1^ in patients with SCD is accompanied by increased cardiac volumes, likely due to chronic anaemia, but is rarely related to a raised PVR_echo_. To confirm this and to establish the contribution of elevated pulmonary venous pressures, we undertook right heart catheterization in 18 SCD patients with TRV ≥2.5 m.s^-1^. Echocardiography was repeated on the day of catheterization; 2 patients had TRV <2.5 m.s^-1^ on this occasion. Patient clinical characteristics were similar to the overall group of 44 patients with TRV ≥2.5 m.s^-1^.

S3 Table shows the invasive haemodynamic and non-invasive data for individual patients. The mean PAP was 22±7 mmHg (range 12–37 mmHg), mean PCWP 13±4 mmHg (range 6–20 mmHg) and mean cardiac output 8 L.min^-1^ (range 4–12 L.min^-1^). 8 patients (44%) had PCWP ≥15 mmHg, indicative of pulmonary venous hypertension. 13 patients (72%) had significant parenchymal lung disease on computed tomography, including the 1 patient who met diagnostic criteria for pulmonary arterial hypertension. All 15 patients who had lung function tests showed abnormal results.

**Fig 2:**
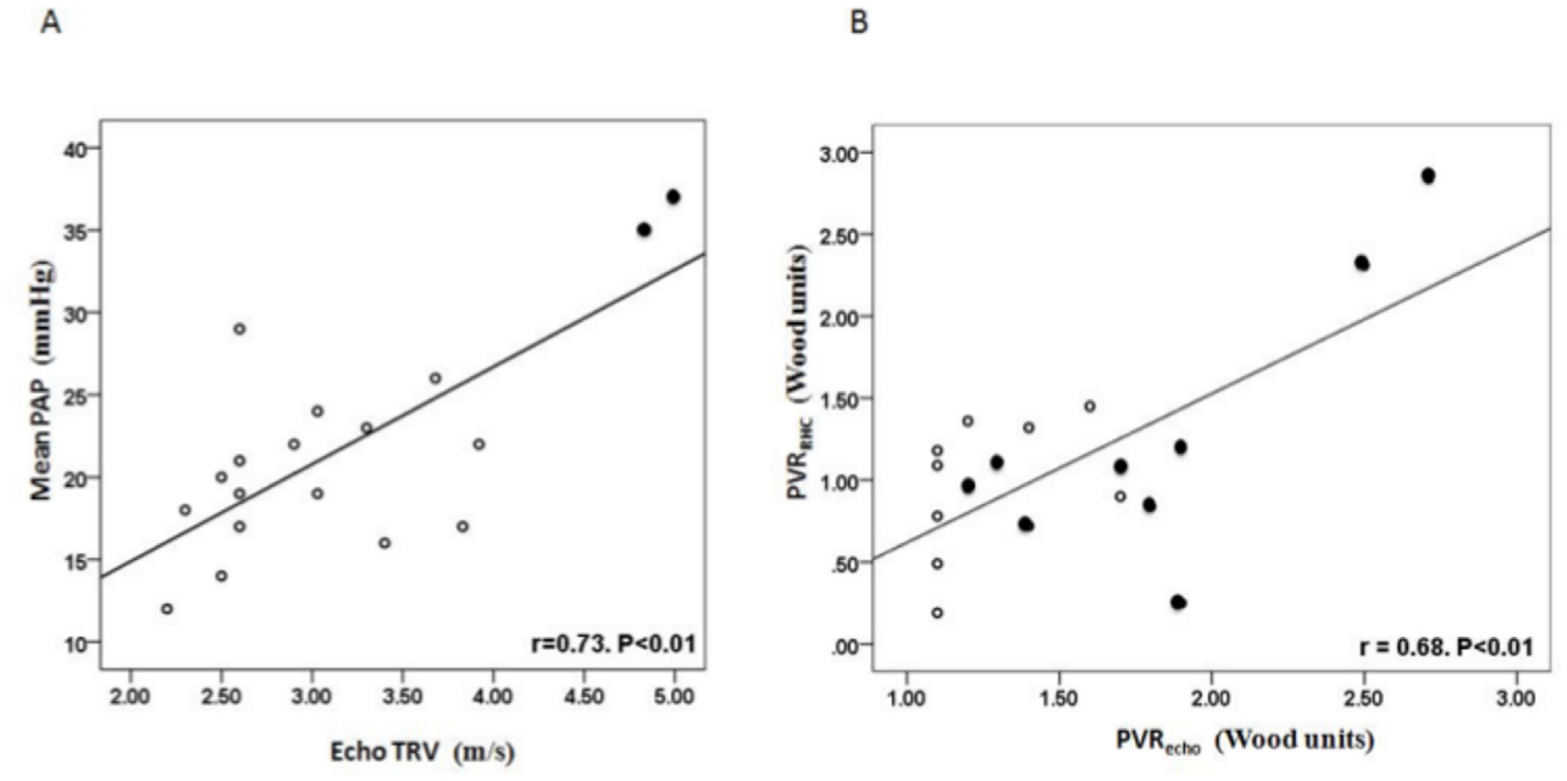
Correlation between echocardiographic estimates and invasive values of pulmonary haemodynamic parameters. A) TRV versus mean PAP; B) PVR_echo_ versus PVR_RHC_. In A, solid symbols denote patients with PVR_echo_ ≥ 2 Wood units. In B, solid symbols denote patients with TRV ≥ 3 m/s.

Fig 2A shows the association between TRV and mean PAP. Although there was a significant correlation, only 4 of 18 patients had a mean PAP ≥25 mmHg, of whom 3 had a PCWP ≥15 mmHg. Therefore, using TRV ≥ 2.5 m.s^-1^ greatly overestimated PAP and only 1 patient met diagnostic criteria for pulmonary arterial hypertension. Fig 2B shows the association between PVR_echo_ and invasively measured PVR (PVR_RHC_). No patient had a PVR_RHC_ >3 Wood units (diagnostic cut-off for pulmonary arterial hypertension). The 2 patients with PVR_RHC_ between 2–3 Wood units had PVR_echo_ values in the same range and had TRV >4 m.s^-1^. None of the 16 patients with PVR_echo_ <2 Wood units had a PVR_RHC_ over this value; i.e. no patients with even physiologically elevated PVR were missed by using a conservative PVR_echo_ cut-off of 2 Wood units.

## Discussion

The main findings of this study in stable SCD outpatients are that: (a) although >35% of patients have TRV ≥2.5 m.s^-1^, there is little evidence of haemodynamically significant pulmonary hypertension as assessed by RV function and non-invasive PVR_echo_; (b) patients with raised TRV have elevated cardiac output, biventricular dilatation and 3D features of volume overload, likely to be related to chronic anaemia. Therefore, the high-output state of SCD rather than primary abnormalities of the pulmonary microvasculature may be the major driver of abnormal pulmonary haemodynamics. To our knowledge, this is the first prospective study to use comprehensive 2D and 3D echocardiography to evaluate the complex cardiopulmonary physiology of SCD outpatients.

The prevalence and role of pulmonary hypertension in the pathophysiology and outcome of SCD are controversial. Reports by Gladwin [4,21] and others [7,8], based primarily on TRV as a marker for pulmonary hypertension, suggested that this was a substantial problem and that targeting the pulmonary microvasculature might be of therapeutic value. It was proposed that chronic haemolysis impairs vascular nitric oxide bioactivity and leads to pulmonary vasoconstriction and pulmonary hypertension in SCD [4]. However, more recent studies that performed systematic cardiac catheterization indicate a much lower (<10%) prevalence of pulmonary hypertension, with post-capillary hypertension predominating over arterial pulmonary hypertension [5,11]. However, it remains unclear whether SCD patients can usefully be evaluated by echocardiography and what the basis is for the elevated TRV values in a large proportion of patients.

Our study confirms the high prevalence of elevated TRV in unselected SCD outpatients but, consistent with previous work [5], we find a very low rate of pulmonary hypertension. Importantly, our study used non-invasive methodology to assess patients and did not exclude any subjects on the basis of disease severity or complications. We found that very few patients had elevated PVR_echo_ and, furthermore, there was no evidence of significant RV dysfunction as assessed by ejection fraction or TAPSE. In fact, SCD patients with TRV ≥2.5 m.s^-1^ had more hyperdynamic RV function than those with lower TRV values indicating an absence of RV pressure overload. These findings were confirmed in the subgroup of patients with TRV ≥2.5 m.s^-1^ who underwent invasive assessment; only 1 patient met the diagnostic criteria for pulmonary arterial hypertension. It should be noted that the thermodilution method may underestimate cardiac output in patients with significant TR and high flow but this would, if anything, result in an overestimation of the PVR.

We then wished to establish the reasons for elevated TRV values in a high proportion of SCD patients. We found that haemoglobin concentration was an independent determinant of TRV ≥2.5 m.s^-1^ but markers of haemolysis (e.g. reticulocyte count, lactate dehydrogenase) were not independent predictors, arguing against haemolysis, nitric oxide consumption and consequent increase in PVR as a potential mechanism. Chronic anaemia in SCD is known to be accompanied by an increase in LV volumes and mass [21–23], and we found that patients with elevated TRV had higher LV and RV volumes than those with TRV <2.5 m.s^-1^. 3D echocardiography not only allowed more accurate estimation of volumes than 2D echocardiography [17] but also enabled calculation of the LV sphericity index. This showed that patients with elevated TRV had globular LV remodelling (a higher sphericity index), suggestive of a volume-overloaded LV due to the high-output state associated with chronic anaemia. We assessed whether LV systolic function was impaired in patients with enlarged ventricles, which could potentially promote adverse remodelling and dilatation. However, systolic function assessed by LV strain, ejection fraction and stroke work appeared well preserved, again pointing to a high-output state and volume overload as the major driver of ventricular dilatation. A high-output state and LV volume overload may contribute to the elevated TRV—which is used as a non-invasive estimate of PAP—through at least two mechanisms. Firstly, since PAP is directly dependent on the product of flow and microvascular resistance, an elevated cardiac output (flow) *per se* may lead to elevated pressures if PVR is unchanged. Secondly, LV volume overload may lead to elevated pulmonary venous pressures and resistance. We found evidence for both mechanisms in that cardiac index was increased in SCD patients and a high proportion (44%) of those who underwent cardiac catheterization had an elevated PCWP. Taken together, these results strongly suggest that a high-output state *per se* is the major driver of abnormal pulmonary haemodynamics, both through direct (flow) and indirect (LV overload) mechanisms. The majority of our patients (67%) had the haemoglobin SS genotype, with only 18% haemoglobin SC and 15% haemoglobin S-beta thalassaemia, and so no systematic comparison among genotypes was undertaken. However, the results in the haemoglobin SS group reflected those in the total cohort. The finding in multivariable analyses that haemoglobin concentration was the main independent determinant of both TRV and LVEDV suggests that any difference between genotypes may be related mainly to the haemoglobin concentration and associated severity of high-volume state.

In this study, we used a non-invasive echocardiography-based estimate of PVR (i.e. PVR_echo_) that has been validated in a non-SCD population [13]. This estimate, while simple to use, does not take filling pressures into account and so may be regarded more an estimate of total pulmonary resistance than pulmonary arterial resistance [24]. This may explain why PVR_echo_ tended to overestimate PVR_RHC_, with SCD patients known to often have raised LV filling pressures [25]. However, such overestimation would reduce the likelihood of false negative results and indeed no patients with PVR_RHC_ >2 Wood units were missed by the use of PVR_echo_. Therefore, our results suggest that PVR_echo_ may be a useful estimate of PVR in SCD.

Other factors, including hypoxia, pulmonary embolism and parenchymal lung disease, may also contribute to abnormal pulmonary haemodynamics in SCD. Our cohort of unselected SCD outpatients illustrates the common occurrence of all these conditions. The underlying mechanism(s) of abnormal pulmonary haemodynamics are of significant therapeutic relevance. Agents such as endothelin receptor antagonists and phosphodiesterase V inhibitors may be effective in pulmonary arterial hypertension where an increased PVR is the major pathogenic factor [20]. However, they would not be expected to be of benefit when the PVR is relatively low. In theory, therapies that target chronic anaemia, hypoxia, pulmonary venous congestion and pulmonary embolism (such as hydroxyurea, domiciliary oxygen, diuretics and anticoagulation) may be more beneficial, although randomised clinical trials to test this are required.

The evaluation of cardiopulmonary haemodynamics and its contribution to disease pathophysiology and outcome in SCD is complex. Invasive right heart catheterization remains the gold-standard for accurate measurement of pulmonary vascular pressures and resistance, but comprehensive non-invasive evaluation by echocardiography can identify a large proportion of patients in whom such invasive assessment may be unnecessary. Our study indicates that 3D analysis of ventricular volumes and sphericity, cardiac index, analysis of RV function, and estimation of PVR_echo_ are especially useful in this population. A limitation of our study is that we did not have detailed data on biomarkers (e.g. NT-proBNP) or objective functional capacity in the study population, which would be valuable in the overall evaluation of these patients. In conclusion, this study suggests that a dominant factor that contributes to abnormal pulmonary haemodynamics in stable SCD is the elevated cardiac output and volume overload state associated with chronic anaemia.

## Supporting Information

S1 Table LV strain. (DOCX)

S2 Table Independent determinants of TRV ≥2.5 m/s and PVR_echo_ ≥2 Wood units. Variables included in the model were: age, gender, history of pulmonary embolus, haemoglobin concentration, LDH level, proteinuria, and cardiac index.
(DOCX)

S3 Table Individual invasive pulmonary hemodynamic and non-invasive parameters in 18 SCD patients. TPG, transpulmonary gradient, ILD, interstitial lung disease.
(DOCX)
