# Supplementary material for: Pulmonary Haemodynamics in Sickle Cell Disease Are Driven Predominantly by a High-Output State Rather Than Elevated Pulmonary Vascular Resistance: A Prospective 3-Dimensional Echocardiography/Doppler Study
Source: PLoS One. 2015 Aug 13;10(8):e0135472. doi: 10.1371/journal.pone.0135472 (PMC4535955; doi:10.1371/journal.pone.0135472)
Supplement: S1 Table — (DOCX) [file pone.0135472.s001.docx]

**S1a Table.** LV longitudinal strain.

|  | **Controls**  **(n=13)** | **SCD**  **(n=22)** | **P value** |
| --- | --- | --- | --- |
| Basal anterior lateral, % | -26±11 | -22±7 | 0.32 |
| Basal interventricular septum, % | -14±5 | -15±6 | 0.61 |
| Mid-anterior lateral, % | -15±6 | -12±5 | 0.17 |
| Mid-interventricular septum, % | -16±5 | -15±4 | 0.61 |
| Apical anterior lateral, % | -11±5 | -18±3 | 0.56 |
| Apical interventricular septum, % | -19±8 | -20±4 | 0.40 |

**S1b Table.** LV circumferential strain.

|  | **Controls**  **(n=13)** | **SCD**  **(n=22)** | **P value** |
| --- | --- | --- | --- |
| Basal septal, % | -18±4 | -19±5 | 0.67 |
| Basal inferior lateral, % | -20±6 | -17±4 | 0.24 |
| Basal anterior, % | -14±5 | -19±3 | 0.07 |
| Basal inferior, % | -15±3 | -16±3 | 0.16 |
| Basal anterior lateral, % | -17±5 | -17±4 | 0.89 |
| Basal inferior septal, % | -19±4 | -19±4 | 0.80 |

**S1c Table.** LV radial strain.

|  | **Controls**  **(n=13)** | **SCD**  **(n=22)** | **P value** |
| --- | --- | --- | --- |
| Basal anterior septal, % | 23±6 | 26±14 | 0.55 |
| Basal inferior lateral, % | 27±16 | 28±10 | 0.83 |
| Basal anterior, % | 24±10 | 19±15 | 0.56 |
| Basal inferior, % | 24±10 | 22±12 | 0.78 |
| Basal anterior lateral, % | 18+6 | 21±18 | 0.67 |
| Basal inferior septal, % | 15±8 | 21±6 | 0.57 |
