# Supplementary material for: Pulmonary Haemodynamics in Sickle Cell Disease Are Driven Predominantly by a High-Output State Rather Than Elevated Pulmonary Vascular Resistance: A Prospective 3-Dimensional Echocardiography/Doppler Study
Source: PLoS One. 2015 Aug 13;10(8):e0135472. doi: 10.1371/journal.pone.0135472 (PMC4535955; doi:10.1371/journal.pone.0135472)
Supplement: S2 Table — Variables included in the model were: age, gender, history of pulmonary embolus, haemoglobin concentration, LDH level, proteinuria, and cardiac index. (DOCX) [file pone.0135472.s002.docx]

**S2 Table. Independent determinants of TRV ≥2.5 m/s and PVR_echo_ ≥2 Wood units.** Variables included in the model were: age, gender, history of pulmonary embolus, haemoglobin concentration, LDH level, proteinuria, and cardiac index.

|  | ***Standardised β*** | **P value** |
| --- | --- | --- |
| **Determinants of TRV ≥2.5 m/s** | | |
| Hemoglobin concentration | − 0.46 | <0.001 |
| History of pulmonary embolism | 0.23 | 0.02 |
| **Determinants of PVR_echo_  ≥2 Wood units** | | |
| Pulmonary embolism | 0.29 | 0.01 |
| Age | 0.23 | 0.03 |
