# Supplementary material for: Pulmonary Haemodynamics in Sickle Cell Disease Are Driven Predominantly by a High-Output State Rather Than Elevated Pulmonary Vascular Resistance: A Prospective 3-Dimensional Echocardiography/Doppler Study
Source: PLoS One. 2015 Aug 13;10(8):e0135472. doi: 10.1371/journal.pone.0135472 (PMC4535955; doi:10.1371/journal.pone.0135472)
Supplement: S3 Table — TPG, transpulmonary gradient, ILD, interstitial lung disease. (DOCX) [file pone.0135472.s003.docx]

| **Patient** | **Hb (g/dL)** | **LDH (IU/L)** | **LVEDVI (mL/m^2^)** | **LV E/E'** | **TRV (m/s)** | **TAPSE (cm)** | **PVR_echo_ (Wood U)** | **CO (L/min)** | **Mean PAP (mmHg)** | **PCW (mmHg)** | **TPG (mmHg)** | **PVR_RHC_ (Wood U)** | **ILD on CT** |
| --- | --- | --- | --- | --- | --- | --- | --- | --- | --- | --- | --- | --- | --- |
| 1 | 7.5 | 489 | 81 | 8.4 | 2.6 | 2.3 | 1.2 | 5.87 | 19 | 11 | 8 | 1.36 | Yes |
| 2 | 8 | 272 | 78 | 5.47 | 4.84 | 1.8 | 2.71 | 8.8 | 35 | 10 | 25 | 2.84 | Yes |
| 3 | 8.1 | 381 | 96 | 12.89 | 3.68 | 2.3 | 1.8 | 7.14 | 26 | 20 | 6 | 0.84 | Yes |
| 4 | 8.1 | 280 | 76 | 8.64 | 2.3 | 2.9 | 1.1 | 7.73 | 18 | 12 | 6 | 0.78 | No |
| 5 | 8.4 | 456 | 68 | 4.2 | 2.6 | 2.4 | 1.1 | 10.53 | 17 | 15 | 2 | 0.19 | No |
| 6 | 7.6 | 380 | 118 | 13.5 | 5 | 2.3 | 2.5 | 9.1 | 37 | 16 | 21 | 2.31 | Yes |
| 7 | 8.1 | 508 | 96 | 7.9 | 3.4 | 2.3 | 1.9 | 12 | 16 | 13 | 3 | 0.25 | Yes |
| 8 | 7.1 | 697 | 84 | 11.08 | 2.9 | 1.7 | 1.7 | 6.63 | 22 | 16 | 6 | 0.9 | Yes |
| 9 | 9.6 | 353 | 75 | 7.53 | 2.6 | 2.5 | 1.6 | 4.13 | 21 | 15 | 6 | 1.45 |  |
| 10 | 8 | 363 | 56 | 5.8 | 3.83 | 2 | 1.7 | 6.52 | 17 | 10 | 7 | 1.07 | Yes |
| 11 | 6.7 | 467 | 53 | 5.08 | 2.6 | 2.2 | 1.1 | 7.63 | 29 | 20 | 9 | 1.18 | Yes |
| 12 | 8.3 | 422 | 64 | 7.44 | 2.5 | 2.3 | 1.1 | 7.32 | 14 | 6 | 8 | 1.09 | Yes |
| 13 | 7 | 495 | 90 | 6.1 | 3.92 |  | 1.9 | 7.53 | 22 | 13 | 9 | 1.2 | Yes |
| 14 | 5.1 | 422 | 111 | 9.5 | 3.03 | 2.2 | 1.4 | 6.97 | 19 | 14 | 5 | 0.72 | Yes |
| 15 | 8.1 | 638 | 68 | 6 | 2.2 | 2.7 | 1.1 | 6.17 | 12 | 9 | 3 | 0.49 | No |
| 16 | 9.2 | 417 | 79 | 10.8 | 2.5 | 2.1 | 1.4 | 8.35 | 20 | 9 | 11 | 1.32 | Yes |
| 17 | 6.5 | 695 | 76 | 10.08 | 3.3 | 2.7 | 1.3 | 7.23 | 23 | 15 | 8 | 1.11 | Yes |
| 18 | 6.7 | 439 | 92 | 8.9 | 3.03 | 3.1 | 1.2 | 7.33 | 24 | 17 | 7 | 0.95 | No |

**S3 Table.** Individual invasive pulmonary hemodynamic and non-invasive parameters in 18 SCD patients. TPG, transpulmonary gradient, ILD, interstitial lung disease.
